# Does size really matter? A sensitivity analysis of number of seeds in a respondent-driven sampling study of gay, bisexual and other men who have sex with men in Vancouver, Canada

**DOI:** 10.1186/s12874-016-0258-4

**Published:** 2016-11-16

**Authors:** Nathan John Lachowsky, Justin Tyler Sorge, Henry Fisher Raymond, Zishan Cui, Paul Sereda, Ashleigh Rich, Eric A. Roth, Robert S. Hogg, David M. Moore

**Affiliations:** 1 Epidemiology & Population Health, British Columbia Centre for Excellence in HIV/AIDS, 608-1081 Burrard Street, Vancouver, V6T 1Y6 Canada; 2 School of Public Health and Social Policy, Faculty of Human and Social Development, University of Victoria, Victoria, Canada; 3 University of California San Francisco, San Francisco, USA; 4 San Francisco Department of Public Health, San Francisco, USA; 5 Department of Anthropology, University of Victoria, Victoria, Canada; 6 Faculty of Health Science, Simon Fraser University, Burnaby, Canada; 7 Faculty of Medicine, University of British Columbia, Vancouver, Canada

**Keywords:** HIV/AIDS, Men who have sex with men, Respondent-driven sampling, Sensitivity analysis

## Abstract

**Background:**

Respondent-driven sampling (RDS) is an increasingly used peer chain-recruitment method to sample “hard-to-reach” populations for whom there are no reliable sampling frames. Implementation success of RDS varies; one potential negative factor being the number of seeds used.

**Methods:**

We conducted a sensitivity analysis on estimates produced using data from an RDS study of gay, bisexual and other men who have sex with men (GBMSM) aged ≥16 years living in Vancouver, Canada. Participants completed a questionnaire on demographics, sexual behavior and substance use. For analysis, we used increasing seed exclusion criteria, starting with all participants and subsequently removing unproductive seeds, chains of ≤1 recruitment waves, and chains of ≤2 recruitment waves. We calculated estimates for three different outcomes (HIV serostatus, condomless anal intercourse with HIV discordant/unknown status partner, and injecting drugs) using three different RDS weighting procedures: RDS-I, RDS-II, and RDS-SS. We also assessed seed dependence with bottleneck analyses and convergence plots. Statistical differences between RDS estimators were assessed through simulation analysis.

**Results:**

Overall, 719 participants were recruited, which included 119 seeds and a maximum of 16 recruitment waves (mean chain length = 1.7). The sample of >0 recruitment waves removed unproductive seeds (*n* = 50/119, 42.0%), resulting in 69 chains (mean length = 3.0). The sample of >1 recruitment waves removed 125 seeds or recruits (17.4% of overall sample), resulting in 37 chains (mean length = 4.8). The final sample of >2 recruitment waves removed a further 182 seeds or recruits (25.3% of overall sample), resulting in 25 chains (mean length = 6.1). Convergence plots and bottleneck analyses of condomless anal intercourse with HIV discordant/unknown status partner and injecting drugs outcomes were satisfactory. For these two outcomes, regardless of seed exclusion criteria used, the crude proportions fell within 95% confidence intervals of all RDS-weighted estimates. Significant differences between the three RDS estimators were not observed.

**Conclusions:**

Within a sample of GBMSM in Vancouver, Canada, this RDS study suggests that when equilibrium and homophily are met, although potentially costly and time consuming, analysis is not negatively affected by large numbers of unproductive or lowly productive seeds.

**Electronic supplementary material:**

The online version of this article (doi:10.1186/s12874-016-0258-4) contains supplementary material, which is available to authorized users.

## Background

Several populations are considered “most at risk” of human immunodeficiency virus (HIV) infection and contribute disproportionately to the epidemic. Such populations include sex workers, injection drug users and gay, bisexual and other men who have sex with men (GBMSM) [1]. Internationally, GBMSM are at a disproportionate risk of HIV infection when compared with other men [2, 3]. In Canada, 2011 prevalence estimates indicated that 33,330 GBMSM were living with HIV (47% of all prevalent cases), with an HIV incidence rate 71 times greater than other men [3, 4]. The HIV epidemic amongst GBMSM is centered within urban contexts. For example in British Columbia, HIV prevalence amongst GBMSM in Metro Vancouver was estimated at 18% in 2009 [4]. Rigorous bio-behavioral surveillance and research with GBMSM is needed, but is hindered by limitations of probability sampling with this population.

Due to a lack of systematic/institutional data collection on relevant behaviors or identities, as well as potential legal barriers and stigma, these GBMSM populations are widely considered to be “hidden” or “hard-to-reach” [1]. Although a population estimate of Vancouver’s GBMSM population has been calculated [5], a complete sampling frame or list of sampling units does not exist for this population. Consequently, it is difficult to generate an unbiased and generalizable sample. While some researchers have found success sampling most at-risk populations through time-location sampling [6], previous research among Vancouver’s GBMSM population identified sub-populations that may not frequent the venues used for sampling [7] and these sub-populations may be underrepresented in time-location sampling. Respondent-driven sampling (RDS) is an increasingly used peer chain-recruitment framework to sample and analyze data from these “hard-to-reach” populations [8, 9]. Globally, there have been over 120 bio-behavioral HIV surveillance studies using RDS methodology, with almost 40 studies focused exclusively on GBMSM [10].

Respondent-driven sampling theory and methodology has been well described in the literature; while not an exhaustive list, the curious reader is referred to the sources cited within this article [8–17]. Currently, there are three RDS-adjustment weighting approaches [8, 9, 13, 17–19]. The first group of estimators, RDS-I (SH), developed in 1997 and later refined in 2004, uses data to make inferences about network characteristics, and then uses those estimates to make inferences about a population parameter point estimate [8, 9]. A second group of estimators, RDS-II (VH), was developed in 2008. These estimators use a Markov chain model to make probability-based calculated estimates directly from the data. As such, these estimators assume that sampling is with-replacement. Additionally, RDS-II (VH) estimation allows for analytical calculation of variance and considers homophily and network size, and not just the latter as for RDS-I (SH) [18]. Using computational simulations to compare these estimators, RDS-II (VH) estimators were found to outperform RDS-I (SH) estimators overall [14, 17]. To prevent the introduction of bias from re-sampling subjects, respondents can only be sampled once in RDS, and as such the assumption of sampling with-replacement is never met with RDS. But when the target population is large enough by comparison to the sample size, known as sample fraction, sampling is said to approximate with-replacement. Finally, a third estimator, RDS-SS, has been developed to address the bias introduced when the assumption of with-replacement sampling is violated, specifically when a large sample fraction exists. The RDS-SS estimator uses successive sampling methodology to approximate parameters and outperforms RDS-II (VH) when sampling is without-replacement. For successive sampling estimation, the target population size must be known. When the sample fraction is small, RDS-II (VH) and RDS-SS estimates converge [13]. If certain assumptions are met, these analyses are said to be asymptotically unbiased [8].

Although the theoretical strengths of RDS are well known, implementation success of RDS varies [10, 12, 20, 21]. Accurate and precise RDS data estimation requires effective implementation of RDS sampling processes [8, 9, 13, 14, 18]. When applied effectively, one particularly important consequence of the RDS process is that the final estimate is not influenced by biases in the initial sampling design; that is, results are not dependent on seed selection. In order for this to occur, there must be enough successive waves for stability on the measured parameter to occur [22]. This can be accomplished by using a small number of seeds, relative to the desired sample size, allowing for enough waves of recruitment before the sample size is met. When larger numbers of seeds are sampled, the desired sample size may be reached with a smaller number of waves and recruitment may be ended before stability of parameters is reached [9, 14, 15, 23]. If this is the case, the use of data provided by unproductive seeds, chosen through biased convenience sampling, may have undue impact on final analysis.

The number of waves required to reach equilibrium is also influenced by the level of homophily, or segregation of sub populations within the target population. If recruits tend to sample from within the same group based on various factors (e.g., age, gender, ethnicity) this indicates a higher level of homophily, which will necessitate more waves to reach stability as there will be a lower probability of recruits sampling from without their group. Furthermore, point-estimate variance increases with increased homophily [8].

It has been suggested that equilibrium will occur within no more than the fourth to sixth wave. [8, 16] While a diagnostic formula to assess if equilibrium has occurred has been developed, it has received some criticism [9, 22].

As an alternative, the use of graphical diagnostics to assess for parameter equilibrium has been proposed. Convergence plots depict a population’s parameter proportion on the y-axis by the number of recruits on the x-axis. As recruitment continues, values will converge on the population estimate with equilibrium indicated by a stabilization of values over remaining recruits, indicating that the sample is not biased by the purposeful selection of seeds over the parameter. Examples of convergence plots are widely available [22]. Convergence plots may hide the effect that individual seeds and their subsequent trees may have on the sample estimate [22]. Bottleneck plots superimpose convergence plots for each individual seed and are useful in assessing if homophily is present. Examples of bottleneck plots are also available [22]. Seed tree plots that converge on or near the population estimate (i.e., one “bottleneck”) is indicative of low homophily. Conversely, different seed tree plots that stabilize on different estimates (i.e., two or more “bottlenecks”) is evidence of homophily [22]. While not statistical hypothesis tests of assumptions these plots can be used to assess visually the properties of population stability and homophily, much like visually assessing QQ-plots to assess normality with regression diagnostics or evaluation of trace plots to assess convergence of Markov chains has become commonplace, for example [24, 25]. These graphical diagnostics can be easily visualized at any stage of an RDS study to examine its success or shortcomings.

While it is analytically desirable to have a small number of seeds and long recruitment chains, this may not always be practical. For example, successive purposeful sampling of unique seeds to access identified sub-populations may be necessary after initial seeds have been selected. Additionally, if recruitment slows, new seeds may be required in order to reach a particular sample size that would have sufficient statistical power to address particular research questions. Indeed, this was our experience implementing an RDS study, and we were therefore curious about the effect of having a larger amount of seeds in our RDS study.

Using data collected from cross-sectional study of GBMSM in Vancouver, British Columbia (BC), we conducted a sensitivity analysis on key study RDS-adjustment weighted point estimates. Our analysis examined the effect that implementing increasingly strict seed exclusion criteria had on point estimates using three different RDS estimators. We hypothesized that when equilibrium and low homophily are graphically observed for a given outcome, RDS point estimates (using any RDS estimator) would remain robust against seed selection bias.

## Methods

The Momentum Health Study of GBMSM in Metro Vancouver, BC, is a cross-sectional RDS study with subsequent semi-annual prospective follow-up. The scale-up of highly active antiretroviral therapy (HAART) in BC through a policy of Treatment as Prevention may affect HIV sexual risk behaviour as mediated by increasing use of soft and hard drugs (including injection and non-injection drugs) [26]. If this risk compensation is substantial, then the HAART scale-up might not bring about a decline in HIV incidence in the GBMSM population. The overall study therefore aims to detect significant but small changes in HIV sexual risk and drug-taking behaviour over the course of the 4 years of follow-up.

### Study population

Participants were recruited into the Momentum Health Study. Eligibility criteria were identify as a man, report recent sex (past 6 months) with another man, be aged ≥ 16 years of age, live in Metro Vancouver, and be able to complete a questionnaire in English. Baseline cross-sectional data were collected between February 2012 and February 2014.

### Recruitment and study procedures

After conducting formative research using community mapping to identify GBMSM characteristics in Vancouver [7] participants were recruited using RDS. Initially, 30 seeds were selected purposively from our formative work, community agency and study team contacts, and our community advisory board with consideration to diversity in terms of age, ethnicity, and HIV status. Seeds were trained in peer recruitment in-person by a research assistant and provided with up to 6 paper or electronic coupons to recruit other GBMSM from their networks. An additional 89 seeds were added to promote further recruitment success of sample size targets, which were additionally recruited using advertisements on popular online social/sexual networking platforms popular amongst GBMSM.

All study subjects were asked to complete a computer assisted, self-administered questionnaire collecting data on demographics, sexual behavior and substance use. A nurse-administered questionnaire and clinical visit was conducted, which included a rapid point-of-care HIV test. Participants received a $50 Canadian dollars (CAD) honorarium as participation incentive. Participation incentive could be either paid in cash or redeemed for a semi-annual prize draw entry for travel ($2,000 value) or monthly prize draw entry for an electronics gift card ($250 value). Participants were also provided a $10 CAD recruitment incentive for each successful participant that completed the study protocol.

### Outcome variables

To describe our sample we include basic demographic variables. Sexual identity was determined as gay or bisexual/other. Age was categorized as 18–29, 30–44 or ≥ 45 years old. Race/ethnicity was self-identified as Caucasian, Asian, Indigenous, or other. Participant annual income was categorized as < $30,000, $30,000-$60,000 or ≥ $60,000 CAD. We also determined if participants had a regular partner at the time of survey, and the number of male anal sex partners respondents had in the past six months, categorized upon quartiles as 0, 1–2, 3–6, or ≥7 partners.

Our sensitivity analysis focuses on three key variables: 1) HIV serostatus (HIV-negative or HIV-positive); 2) any “high risk sex”, which was defined as any condomless anal intercourse in the past 6 months with an HIV-discordant or status unknown partner; and 3) any injection drug use (excluding steroids) in the past six months. HIV serostatus was determined using a nurse-administered point-of-care HIV test (Insti^TM^ Rapid HIV-1/HIV-2 test, Biolytical Laboratories, Richmond, Canada) with subsequent typical confirmatory testing for reactive or indeterminant results at the local public health laboratory, or for study participants who self-reported as being HIV positive, confirmation of their HIV status with a previous laboratory report. All newly diagnosed participants were referred to care.

### Sample size

Prior research in Vancouver approximated a prevalence of condomless anal intercourse with a sero-discordant partner of unknown HIV status of 20% among GBMSM, and the prevalence of any hard or soft drug use within the two hours prior to anal intercourse, a possible predictor of high-risk sex, of 26% [4]. In order to detect a significant difference of +/− 8.6% with a power of 0.9 at *p* = 0.05 of condomless anal intercourse with a sero-discordant or unknown HIV status partner and an odds ratio of 1.52 or larger for the effect of drug-use around sex on having risky sex with a power of 0.8 at *p* = 0.05, we calculate a minimum required sample size of 560 after excluding a planned 30 seeds.

### Statistical analysis

Descriptive analysis was used to calculate crude and RDS-adjustment weighting point-estimates and 95% confidence intervals (CI) of outcome variables. Any missing data were treated as non-response and coded as such for all analyses. RDS estimates were conducted with functions RDS-I (SH) [8, 9], RDS-II (VH) [18] and RDS-SS [13]. For RDS-II (VH) weights, a participant’s network size was determined using the following questions asked on the computer-assisted self-interview questionnaire: “Of the GBMSM you know in the Vancouver area and whom you have seen or spoken to in the past month, how many do you know comfortably enough to give a study voucher inviting their participation (in the study)?” RDS-SS estimates, which require the population size be known, assumed a population size estimate of 33,960 GBMSM in Vancouver as previously described [5]. To conduct sensitivity analysis, we used various sample cuts, starting with all participants and subsequently removing unproductive seeds (0 recruitment waves), chains of ≤ 1 recruitment waves, and chains of ≤ 2 recruitment waves.

A simulation analysis was performed to compare estimates and variances of outcome variables between the three RDS-weighting functions used. At random, a sub-sample of seed trees was selected from the overall sample and estimates were calculated using the three chosen RDS-weighting functions. This process was repeated for 100 sub-samples. Pair-wise comparisons between each estimate were performed using a level of significance of α = 0.05. If the difference in estimates tended to be always >0 or <0 that would indicate that one method tended to produce greater or smaller estimates than the other.

Data were cleaned using SAS 9.4 and analysis and plots were done using RDS Analyst 0.52 [27]. Diagnoses were observed visually with convergence plots and bottleneck plots, with functions ‘convergence.plot’ and ‘bottleneck.plot’ respectively [22].

## Results

### RDS characteristics

**Fig. 1:**
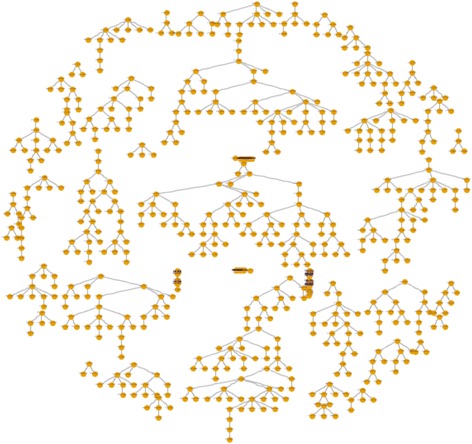
Momentum Health Study recruitment tree. Nodes represent one study recruit. Seeds are represented by superior terminus nodes

Overall, 719 participants were recruited, which included 119 seeds (16.6% of overall sample) and a maximum of 16 recruitment waves (mean recruitment chain length = 1.75 waves). The recruitment tree depicting sample networks is presented in Fig. 1. The removal of unproductive seeds (*n* = 50, 42.0% of sample seeds, 7.0% of overall sample), those that did not recruit any participants, left 69 productive seeds that resulted in at least one recruitment wave. For this sub-sample, the mean recruitment chain length increased to 3.01 waves. By removing seeds that only produced one recruitment wave (*n* = 125 seeds or recruits, 17.4% of overall sample), 37 moderately productive seeds remained, with the mean recruitment chain length increasing to 4.76 waves. Finally, removal of seeds that only produced two recruitment waves (*n* = 182 seeds or recruits, 25.3% of overall sample) left 25 highly productive seeds, with a mean recruitment chain length of 6.08 waves. The wave-length characteristics of each sub-sample are summarized in Table 1.

**Table 1:** Wave-length characteristics of four differing sample restrictions based on seed productivity

	Overall (*n* = 719)	>0 Wave (*n* = 669)	>1 Wave (*n* = 594)	>2 Waves (*n* = 537)
Seeds^a^ *n*(% of sample remaining)	119 (16.6%)	69 (10.3%)	37 (6.2%)	25 (4.7%)
Wave length mean (median)	1.75 (1.00)	3.01 (2.00)	4.76 (3.00)	6.08 (5.00)
Wave length range	0–16	1–16	2–16	3–16

^a^Number of seeds corresponds with number of chains

### Sample demographics

Our sample of 719 GBMSM contained 612 (85.1%) men that identified as gay. There was a reasonably even distribution of age among our sample, ranging from 18 to 74 years with a median of 33 years (interquartile range 26–47 years). The majority of our sample consisted of respondents who identified their race/ethnicity as Caucasian (*n* = 539, 75.0%). Most respondents reported earning less that $30,000/year (*n* = 457, 63.6%). Our sample consisted of a majority of respondents that did not report having a current partner (*n* = 446, 62.3%) and 629 (87.6%) respondents reported more that one anal sex partner in the past 6 months. Descriptive crude variables of our sample can be found in Table 2.

**Table 2:** Momentum Health Study sample demographics

	*n*	Crude %
Sexual identity		
Gay	612	85.1
Bisexual/Other	107	14.9
Age (years)		
18–29	275	38.3
30–44	233	32.4
≥ 45	211	29.5
Race/Ethnicity		
White	539	75.0
Asian	72	10.0
Indigenous	50	7.0
Other	58	8.1
Income (CAD)		
< $30,000	457	63.6
$30,000–60,000	182	25.3
> $60,0000	80	11.1
Regular sex partner		
Yes	273	37.0
No	446	62.3
Number of male anal sex partners in past 6 months		
0	89	12.4
1–2	226	31.5
3–6	208	30.0
≥ 7	195	27.2

### Diagnostic plots

**Fig. 2:**
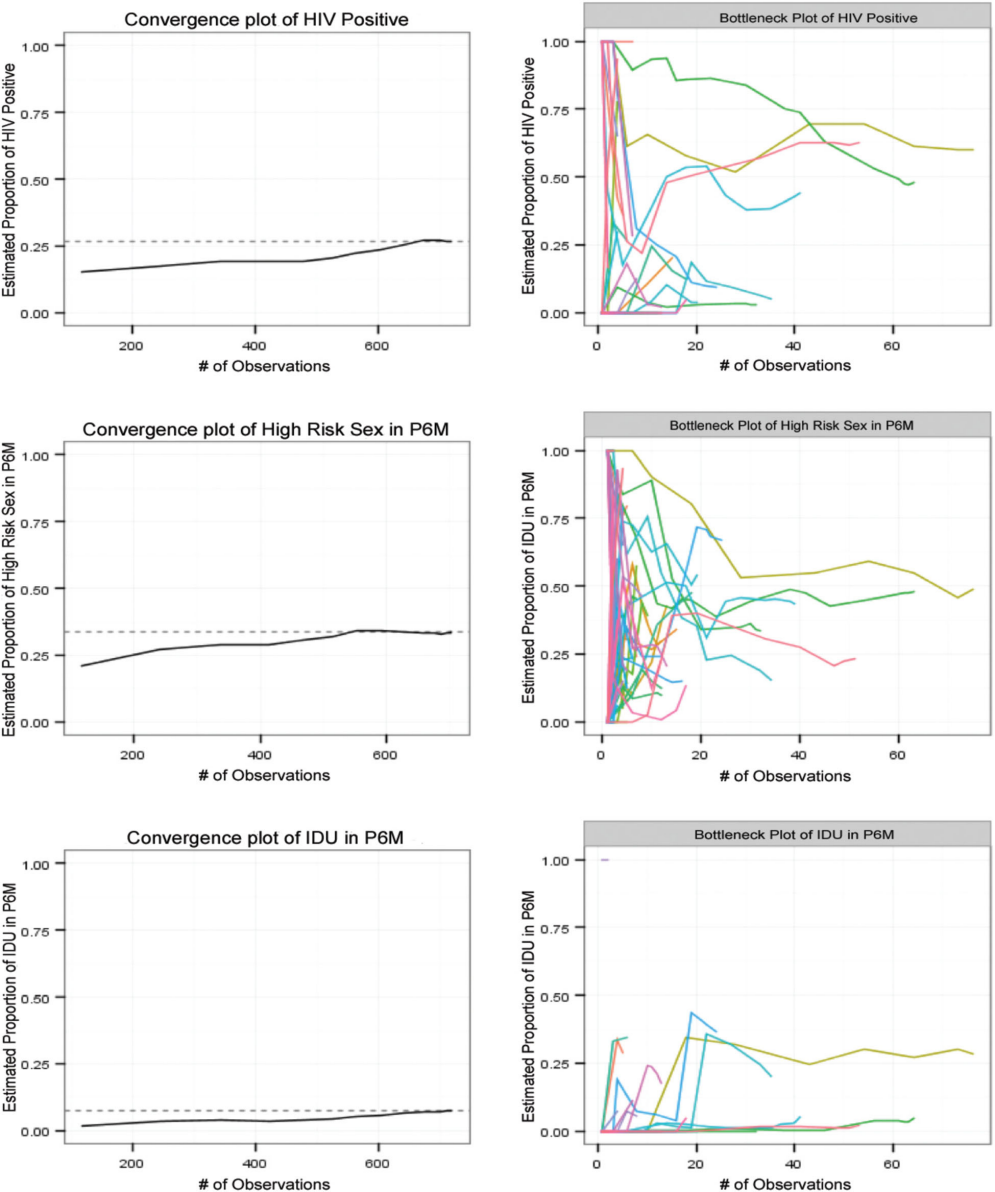
Convergence and bottleneck plots of three key outcome variables of the Momentum Health Study. Plot y-axes represent proportions of participants that answered “yes” to the parameter, x-axes are number of participants. P6M = past six months, IDU = injection drug use, excluding steroids

Diagnostic plots were produced at the end of recruitment and data collection. Convergence plots of high-risk sex and injection drug use showed that both variables converged on the population estimate (Fig. 2). Additionally, the bottleneck plots for both variables appeared to converge on the point estimate, suggesting low homophily (Fig. 2). Contrastingly, the convergence plot of the HIV-positive serostatus variable showed very late convergence of sample results on the population estimate. Furthermore, this bottleneck plot showed two divergent estimates, suggestive of sample homophily. Analytically, convergence was found to have occurred by the 10th wave (*n* = 691 including seeds) at a level of 0.01 homophily for all three key outcome variables (data not shown).

### Sensitivity analysis

Based on the various sample restrictions excluding seeds and their recruitment chains stepwise by productivity, Table 3 provides estimates for three key study outcomes. For high risk sex and injection drug use, within each sample cut, crude, RDS-I (SH), RDS-II (VH) and RDS-SS adjusted estimates of proportions fell within each estimates’ confidence interval. Additionally, we find that for outcome HIV-positive serostatus, within each sample cut, crude estimates did not fall within the RDS-I (SH) confidence interval, but did fall within RDS-II (VH) and RDS-SS confidence intervals.

**Table 3:** Three key study outcomes using various sample cuts and RDS-weights

		*n*	Crude %	RDS I % (95% CI)	RDS II % (95% CI)	RDS SS % (95% CI)
HIV-positive serostatus (vs. HIV-negative)	Overall	199	27.7	22.5 (19.4–25.6)	26.7 (20.7–32.7)	26.7 (20.7–32.7)
	>0 Wave	189	28.3	22.9 (19.7–26.2)	27.7 (21.5–33.9)	27.7 (21.4–34.1)
	>1 Wave	178	30.0	24.2 (20.6–27.7)	29.3 (22.6–36.0)	29.4 (22.6–36.1)
	>2 Wave	161	30.0	23.8 (20.1–27.5)	29.1 (22.1–36.1)	29.1 (22.1–36.2)
Any high risk sex in past 6 months (vs. none)	Overall	262	37.3	34.9 (31.1–38.8)	33.6 (27.6–39.6)	33.7 (27.7–39.7)
	>0 Wave	251	38.4	35.5 (31.5–39.5)	35.2 (28.9–41.5)	35.3 (29.0–41.6)
	>1 Wave	221	38.0	36.0 (31.7–40.3)	35.8 (29.3–42.4)	35.8 (29.2–42.4)
	>2 Wave	203	38.7	37.0 (32.4–41.5)	36.4 (29.5–43.3)	36.5 (29.6–43.3)
Injected any drugs in past 6 months (vs. none)	Overall	61	7.1	8.8 (5.5–12.1)	7.3 (3.9–10.7)	7.3 (3.9–10.7)
	>0 Wave	51	7.6	9.1 (5.8–12.4)	8.1 (4.4–11.8)	8.1 (4.3–11.9)
	>1 Wave	48	8.1	8.7 (5.7–11.7)	8.0 (4.7–11.3)	8.0 (4.7–11.3)
	>2 Wave	43	8.0	8.7 (5.5–11.9)	8.3 (4.8–11.8)	8.3 (4.8–11.9)

Simulation analysis did not find any significant differences between RDS estimators using paired comparisons. Differences between RDS-II (VH) and RDS-SS estimators were 0.0, while the absolute differences between RDS-II (VH) and RDS-I (SH) and RDS-SS and RDS-I (SH) were ≥ 1.2. Table 4 shows the results of these analyses, simulation samples and estimates are provided in the supplementary material (Additional file 1).

**Table 4:** Paired comparison of RDS weights for three key study outcomes

	Crude	RDS-I	RDS-II	RDS-SS	RDS-II – RDS-I	RDS-SS – RDS-I	RDS-SS – RDS-II
	HIV-positive serostatus						
Mean % (95% CI)	27.0 (12.3–39.3)	22.2 (10.3–31.9)	25.7 (10.7–36.3)	25.7 (10.7–36.3)	3.4 (−0.9–7.5)	3.4 (−0.9–7.5)	0.0 (−)
*p*-value					0.11	0.11	0.22
	Any high risk sex in past 6 months						
Mean % (95% CI)	37.0 (32.2–41.9)	34.3 (26.7–41.4)	33.1 (25.3–39.4)	33.1 (25.3–39.4)	−1.2 (−2.9–0.3)	−1.2 (−2.9–0.3)	0.0 (0.0–0.1)
*p*-value					0.12	0.12	0.28
	Injected any drugs in past 6 months						
Mean % (95% CI)	6.6 (2.6–11.3)	9.0 (2.3–14.0)	6.7 (1.8–11.7)	6.7 (1.8–11.6)	−2.3 (−3.0–0.0)	−2.3 (−3.0–0.0)	0.0 (−)
*p*-value					0.06	0.06	0.18

## Discussion

Using RDS methodology for a cross-sectional study of 719 GBMSM in Vancouver, BC, our results suggest that point estimates for parameters upon which our sample reached equilibrium with low homophily (e.g., high risk sex and injection drug use) were not effected by the inclusion of unproductive seeds or short recruitment chains in analysis. This was assessed through visualization of diagnostic plots and examination that point estimates calculated fell within the 95% CIs of overall estimates across all RDS-adjustment weighting approaches for various sample restrictions. That crude sample proportions of these parameters fell within all RDS-adjustment weighting approaches’ 95% CIs, within each sample cut based on seed productivity strengthens our conclusion that our estimates are not influenced by potential biases in seed selection. Analytically, we did not find any statistically significant differences between RDS estimators using pair-wise comparison. We conclude that within our sample, when parameter stability and low homophily are met, our analysis is not affected by using large numbers of unproductive seeds, as has been suggested [9, 14, 15, 23]. Although this may seem a costly and time-consuming method of recruitment, we found that we were able to reach our desired sample size by introducing additional seeds into the sample; this allowed us to maintain our sample size, thus limiting variance and preserving statistical power. While these conclusions were based on the relatively large number of seeds used in our analysis, these conclusions may not generalize to studies using more seeds where data from non- and lowly-productive seeds may in fact contribute bias to calculated point estimates. We encourage researchers that depend on a larger proportion of seeds than we present to assess if they will inflict undue bias upon the point estimate.

Contrastingly, when the assumptions of parameter stability and low homophily are violated, such with our HIV serostatus variable, as determined by very late convergence and evidence of two bottlenecks on diagnostic plots, we find some key differences. We find that crude estimates fell within RDS-II (VH) and RDS-SS calculated 95% CIs but not within the RDS-I (SH) calculated 95% CIs across all sample cuts. However, in the context of low number of waves (i.e., with the inclusion of unproductive and less productive seeds) and lower homophily, RDS-II (VH) has been found to outperform RDS-I (SH) [14, 17], and we will therefore lend more trust to RDS-II (VH) estimates when seed bias is present.

When comparing RDS-II (VH) with RDS-SS estimates we found that point estimates, as well as 95% CIs, all fell within 0.1% of each other across all sample cuts for all variables. It has been suggested that RDS-SS estimates can be used to validate the with replacement assumption of RDS-II (VH). Assuming that our Vancouver GBMSM population estimate is robust [5], we conclude that the with replacement sampling assumption is met and that global exhaustion or finite population effects have not introduced bias into our estimates [13].

Finally, our results support previous suggestions that convergence and bottleneck plots are an effective way to determine sample stability and level of homophily [22]. We believe that our estimates are robust for outcome variables that reached equilibrium as evidenced by diagnostic plots, which is supported by prior research [22]. Although we produced these diagnostics at the end of recruitment, we believe that these plots can be easily created and assessed during any stage of sampling to determine if further recruitment is required to reach stability or if further addition of specific unique seeds is required to address sample bottlenecks accounting for low homophily. We feel that our study contributes an empirical “proof of concept” of the diagnostics presented by Gile and colleagues where observational evidence is lacking [17, 19, 22].

Noteably, analytical homophily was observed on all three key outcome variables. This is in contrast to the observed homophily on the HIV serostatus variable determined through the diagnostic bottleneck plot. This may suggest that graphical determination of equilibrium and homophily is better suited to empirical data than simulated data.

To our knowledge, this is the first study to report a sensitivity analysis of varying levels of seed productivity within an RDS study in the literature. Further, we believe this to be the first study within Canada to successfully apply RDS to a GBMSM population. We believe our study contributes empirical evidence to a somewhat novel and increasingly used sampling and analysis methodology where a relative paucity exists.

### Limitations

Respondent-driven sampling with GBMSM populations has been used extensively in non-Western settings, which have unique community-level and societal-level factors in terms of connectedness, acceptance, and stigma. One limitation of our findings is that inferences should not be made to other regions or populations that demonstrate characteristics not consistent with those of our sampled population. Indeed, prior research suggests that population characteristics may vary country to country based on underlying network structure, psychology, behavior, cultural practices, etc. [28, 29] Additional characteristics of our study further limit its generalizability: for example, our findings represent a sample with low homophily, but the inference of these findings to populations with greater homophily on key outcomes may be limited.

Our sensitivity analysis of increasingly removing respondents based on recruitment productivity has led to a reduction in sample size. Inherently, variance will be increased. This limits our use of overlapping 95% CIs, or lack thereof, as an adequate measure of whether or not the differing seed exclusion criteria and choice of estimator made a meaningful impact on estimation. Indeed, RDS variances are shown to be relatively wide already [12, 20]. This was the impetus for us to carry out the simulation analysis for this study.

Additionally, interpretation of our HIV serostatus results should be made with caution due to the potential dependence of our overall analysis on seed selection on this parameter, as assessed through diagnostic plots. Indeed, we suggest interpretation of this variable be limited to our sample cut including only the most productive seeds. We therefore suggest that when stability or homophily are not assessed on a given parameter that final analysis exclude unproductive and lowly productive seeds. This will likely reduce sample size, thus increasing variance and limiting power to detect differences when comparing differing groups or methods of analysis.

A particularly large limitation of all RDS studies is increased variance compared with more traditional data analysis methods, and this applies to the study presented here [12, 20].

As with all observational studies, our analysis may be limited to unobserved selection bias and confounding. Particular to chain-referral sampling methods in general, subpopulations that are not penetrated, or recruited, may exist. While formative assessment attempts to address this by identifying these subpopulations [7, 11], such isolated “out-groups” that are unknown to researchers, based on cultural differences or discriminatory behaviors or perhaps because of a different parameter prevalence, will have led to a form of selection bias. This form of selection bias may still be at play within our study.

The authors view this work, previous analyses [7, 30], and future analyses of the Momentum Health Study as examples of the successful implementation of RDS to derive inferential information of a population without a comprehensive sampling frame. This work, and the body of knowledge cited within, provides support of an emerging method to obtain valid inferences from a non-probability sample, while remaining cautious of its limitations. We encourage those considering the use of RDS to proceed with an understanding of the number of assumptions that must be met for unbiased analysis, and we offer this sensitivity analysis as an example of how to empirically assess some of these assumptions.

## Conclusions

Using diagnostic methods suggested by Gile, Johnston and Salganik [22], for outcomes that have reached parameter stability and within each sample cut, the crude proportions fell within 95% confidence intervals of all RDS-weighted estimates. All RDS-weighted estimates were similar and fell within the 95% confidence intervals of each other on these outcomes. We did not find significant differences between RDS estimators analytically. Furthermore, we find that diagnostic plots are a useful method to assess for equilibrium and homophily within an RDS sample and this is a useful predictor of the validity of descriptive estimates. RDS studies, although potentially costly and time consuming, are not negatively affected by large numbers of unproductive or lowly productive seeds when equilibrium has occurred. These conclusions may not hold true in instances of instability and/or low homophily, as evidenced by the HIV serostatus variable of this study.

## Additional file

Additional file 1: Supplemental Material. (XLSX 823 kb)

## Abbreviations

BC: British Columbia
CAD: Canadian dollar
CI: Confidence interval
GBMSM: Gay, bisexual and other men who have sex with men
HAART: Highly active antiretroviral therapy
HIV: Human immunodeficiency virus
RDS: Respondent-driven sampling
